# Could climate trends disrupt the contact rates between *Ixodes ricinus* (Acari, Ixodidae) and the reservoirs of *Borrelia burgdorferi* s.l.?

**DOI:** 10.1371/journal.pone.0233771

**Published:** 2020-05-29

**Authors:** Natalia Fernández-Ruiz, Agustin Estrada-Peña

**Affiliations:** 1 Department of Animal Health, Faculty of Veterinary Medicine, University of Zaragoza, Zaragoza, Spain; 2 Emerging Zoonoses Research Group, Instituto Agroalimentario de Aragón (IA2), Zaragoza, Spain; Umeå University, SWEDEN

## Abstract

This study addresses the modifications that future climate conditions could impose on the transmission cycles of *Borrelia burgdorferi* s.l. by the tick *Ixodes ricinus* in Europe. Tracking the distribution of foci of a zoonotic agent transmitted by vectors as climate change shapes its spatial niche is necessary to issue self-protection measures for the human population. We modeled the current distribution of the tick and its predicted contact rates with 18 species of vertebrates known to act as reservoirs of the pathogen. We approached an innovative way for estimating the possibility of permanent foci of *Borrelia afzelii* or *Borrelia garinii* tracking separately the expected spatial overlap among ticks and reservoirs for these pathogens in Europe. Environmental traits were obtained from MODIS satellite images for the years 2002–2017 (baseline) and projected on scenarios for the years 2030 and 2050. The ratio between MODIS baseline/current interpolated climatologies (WorldClim), and the ratio between MODIS-projected year 2050 with five climate change scenarios for that year (WorldClim) revealed no significant differences, meaning that projections from MODIS are reliable. Models predict that contact rates between the tick and reservoirs of either *B*. *garinii* or *B*. *afzelii* are spatially different because those have different habitats overlap. This is expected to promote different distribution patterns because of the different responses of both groups of reservoirs to environmental variables. Models for 2030 predict an increase in latitude, mainly in the circulation of *B*. *garinii*, with large areas of expected permanent contact between vector and reservoirs in Nordic countries and central Europe. However, climate projections for the year 2050 predict an unexpected scenario of contact disruption. Though large areas in Europe would be suitable for circulation of the pathogens, the predicted lack of niche overlap among ticks and reservoirs could promote a decrease in permanent foci. This development represents a proof-of-concept for the power of jointly modeling both the vector and reservoirs in a common framework. A deeper understanding of the unanticipated result regarding the year 2050 is needed.

## Introduction

Pathogens transmitted by arthropods have increasing importance in public health. One of the many examples is the expansion of *Aedes albopictus*, a mosquito vector of the etiological agents of Dengue, Zika, and Chikungunya [1]. Although mosquitoes are regarded as the most prominent vectors in human health, ticks are the arthropods that transmit the largest number of zoonotic agents in the Northern Hemisphere [2]. Some of these pathogens are tick-borne encephalitis virus (Flaviviridae), *Ehrlichia* spp., *Anaplasma phagocytophilum*, and bacteria of the genus *Borrelia*, which may cause severe clinical processes in humans [3].

Climate change is one of the factors that shape the possible changes of distribution and seasonality of vectors and the pathogens they transport to areas where they were not previously found, in what is being called (re)emerging foci of zoonoses. Climate change has been defined as, "a statistical phenomenon that describes the average climatic conditions for a region, referring to systematic and generally gradual changes in the trend, which is integrated into random climate fluctuations" [4]. Climate trends drive changes in the geographic distribution and seasonality of ticks, their vertebrate hosts, and the reservoirs of pathogens, shaping the persistence of pathogen foci [5]. Warmer and shorter winters may increase the survival of ticks, the probability of pathogen transmission, and the number of infected reservoirs [6].

Lyme borreliosis is the most frequently recorded tick-borne zoonosis in the Holarctic [7]. Approximately 26 species are recognized in the genus *Borrelia*, some of which are included in the *Borrelia burgdorferi* sensu lato (s.l.) group (Bb). Some of the most important species of the complex are *B*. *burgdorferi* sensu stricto (s.s.), which produces arthritis, polyneuritis, and a skin lesion called erythema migrans; *Borrelia afzelii*, which has special tropism for the skin and can lead to cutaneous lymphadenosis; and *Borrelia garinii*, which generates meningitis and inflammatory lesions in the peripheral nerves [8]. The main vector in Europe is *Ixodes ricinus*, although in other geographic areas other species of Bb can be transmitted by different tick species, such as *Ixodes pacificus* in the western United States, *Ixodes scapularis* in the eastern and central northern United States and southern Canada, or *Ixodes persulcatus*, which is distributed in Eurasia [9]. The incidence of Lyme borreliosis in the Northern Hemisphere is alarming, not only because of the severe clinical picture, but because no harmonized prevention plans are available and vector control still relies on self-protection by the human population. Surveys conducted in Sweden between 1980 and 2008 showed that permanent populations of the tick *I*. *ricinus* had increased in latitude, supporting climate change as one of the most influential factors in its expansion [10]. In addition, both *I*. *ricinus* and the pathogens it transmits are being established at higher altitudes. Systematic sampling conducted in the Krkonoše mountains in the Czech Republic indicated that the limit of altitude distribution for this tick changed from 750 m to up to 1300 m above sea level between 1990 and 2010 [11]. This spatial expansion of permanent tick populations has also been shown in other countries; a comparative study using historical data collected in Norway demonstrated an altitude increase of almost 600 m above sea level and the spread of *I*. *ricinus* into regions near the west coast of the country, where it was traditionally colder than currently [12]. These events do not occur only in Europe, but also in North America, where one of the most important tick species for public health, *I*. *scapularis*, is spreading northward, moving towards Canada and settling in large regions of southern Canada [13].

There is a demand for plausible future patterns of the range of pathogens under conditions of global environmental change, setting priorities for public health. Modeling efforts are commonly addressed to map the expected distribution of ticks based on abiotic descriptions of the environment. However, reports have demonstrated the better performance of jointly modeling both ticks and their vertebrate hosts (or pathogen reservoirs) to capture the probable distribution of health-affecting infectious agents [14]. Few other efforts have exploited process-driven models to capture the life cycle of *I*. *ricinus* [15–17], and only two of them addressed the potential impact of climate trends on the life cycle of *I*. *ricinus* without explicit modeling of the reservoir’s range [16], or assuming its presence according to categories of habitat [17].

*Ixodes ricinus* parasitizes a wide range of vertebrate hosts, though only some of them can be considered reservoirs of Bb. The reservoirs of these bacteria may be birds, rodents, or insectivores. Ticks also use other hosts, such as domestic and wild ungulates, which are not reservoirs, but are essential for tick survival [18]. The different contact rates among ticks, supporting hosts, and reservoirs, exert an important effect on the prevalence of *Borrelia* spp. in ticks and are responsible for the variable local prevalence of the pathogen in ticks [19]. For a focus to persist, there must be (i) appropriate environmental conditions for the survival of the vector, (ii) an adequate density of reservoirs, and (iii) a temporal and spatial overlap between both reservoirs and vector.

The dynamics of these foci, the conditions that propitiate them, and their long-term evolution are yet unanswered but for local scales. This study aims to separately model the spatial probability of the contact rates among *I*. *ricinus*, its hosts, and the most important reservoirs reported for two species of *B*. *burgdorferi* s.l. in Europe, namely *B*. *afzelii* and *B*. *garinii*. The framework is based on a previous development aimed to evaluate the persistence of foci of pathogens through an strict definition of contact rates based on environmental features [20]. Temperature and vegetation variables obtained for the period 2002–2017 are projected to build scenarios for the years 2030 and 2050 to model the probable distribution of hosts, reservoirs, and the tick according to these scenarios, and derive the predicted contact rates. This separate modelling by species and reservoirs seeks to generate basic information on the impact of climate on Lyme borreliosis, addressing the changes in contact rates of the main actors supporting pathogen foci under the expected future climate condition.

## Material and methods

### Background

In this study, we adhered to a strict definition of the environmental niche of the tick *I*. *ricinus*, as well as its reservoirs and hosts [14, 20]. The environmental niche of a pathogen is not spatial, as commonly addressed, but defined as the combination of traits in an area (temperature, humidity) whose intersections allows reservoirs, vectors, and their hosts to survive, generating stable pathogens foci. The niche shapes a probability for the vector, its hosts, and the main reservoirs of Bb to coexist. We use such a definition to define contact rates among ticks, hosts, and reservoirs based on current climate data, and the projected climate for the years 2030 and 2050 based on the land surface temperature (LSTD) and the Normalized Difference Vegetation Index (NDVI). The NDVI is an estimate of the photosynthetic activity of the vegetation, but many studies have utilized this index as a proxy of the relative humidity of an area [21, 22]. In addition, these variables and their annual oscillations are the best descriptors of the environmental niche of arthropod vectors [23]. The expected distribution of each organism has been generated with the actual climate data in the period 2002–2017 recorded by the MODIS series satellites. The climate trend in this baseline period was used to build the scenarios for the years 2030 and 2050.

We adhered to a previous development that promotes the coefficients of a harmonic regression, also known as a Fourier series, representing the value of each pixel for one complete year, as the environmental traits that adequately describe the habitat for ticks [14]. We demonstrated that the use of satellite imagery improves the modeling output compared to interpolated climatologies because satellite records represent measurements on the ground surface, where ticks live [20]. This method uses only six variables (the three first coefficients of the harmonic regression for either LSTD or NDVI) to define the environmental niche. This is an improvement in the model output because it uses variables with ecological significance for the dynamics of the ticks [20] and the absence of over-fitness, a statistical issue that could produce a falsely correct model if too many descriptive variables are used [24].

### Obtaining climate data

To describe the environmental niche of the organisms involved in the transmission of Bb, climate data have been obtained from the MODIS website. Interpolated climate data have not been used because they lack moisture estimates (only precipitation is available), resulting in models of insufficient quality, probably because moisture has a higher performance than precipitation in modelling the distribution of *I*. *ricinus* [25]. Products from the Terra series of satellites (https://modis.gsfc.nasa.gov/data/dataprod/) were used between the years 2002 and 2017. These data have a spatial resolution of 0.05° (approximately 5,600 meters) and correspond to the products MOD11C3 (for LSTD) and MOD13C2 (for the NDVI). We chose these years because data from subsequent years (i.e., after the year 2018) were not available with scientific precision at the time of this study. For the selection, download, and processing of the data corresponding to the target territory, the “MODIStsp” library [26] was used for the R programming environment [27] that allows access to the server API of MODIS. We used monthly data from the mentioned time period between coordinates 18° O and 41.5° E, 30.7° N and 73° N.

### Climate data processing

The MODIS climate dataset was processed in five steps: (i) calculation of the average monthly values for each month in the period 2002–2017 resulting in one year of averaged monthly values, (ii) calculation of the Fourier coefficients that define LSTD and NDVI in the average year for the period 2002–2017, (iii) calculation of the temporal trend of each month in the period 2002–2017, (iv) extrapolation of that monthly trend to the years 2030 and 2050 to obtain the monthly values of LSTD and NDVI for those years, and (v) calculation of the Fourier coefficients for those new scenarios describing the projected climate environments. All of the previous steps were performed separately for the LSTD and NDVI variables. The Fourier series coefficients were calculated using an already developed, published, and open script [14].

The temporal trend of each month was calculated using the “lm” (linear model) function of R [27], taking the slope of the regression line as the indicator of the trend of LSTD or NDVI between 2002 and 2017 for each pixel. The new projected data for each month were obtained by replacing the “x” of the regression line with the new values to be calculated (months of the years 2030 or 2050). After the new monthly values were obtained, Fourier coefficients were calculated for each year.

The linear regression was subjected to a quality control to verify that the modeled climate data were kept within adequate margins of error. Comparing the output of a linear regression producing future values of LSTD and NDVI to the already available scenarios based on interpolated climatologies is complex. Both products differ, in addition to the obvious difference between the temperature recorded at the ground level or at 2 m above the ground. Moreover, interpolated climatologies have no data on NDVI, as it is a satellite-derived product only. We performed a quality control comparing the annual averaged LSTD from MODIS with the average annual temperature in WorldClim at the resolution of 0.05° [28]. We also compared the annual averaged MODIS-derived LSTD projected to the year 2050 to the average annual temperature obtained in five climate scenarios available in WorldClim, for a Representative Concentration Pathway (RCP) of 45 (HadGEM-AO, HadGEM-CC. HadGEM-ES, GFDL-CM3, and GFDL-ESM2G). A RCP is a grenhouse gas concentration (not emissions) trajectory adopted for calculation of future climate scenarios. Next, we carried out an ANOVA to determine significant differences between the ratios of the baseline periods (MODIS *vs* current WorldClim) and those of the year 2050 (MODIS *vs* climate scenarios). If significant differences are not apparent between the two periods, then the satellite-based projections of temperature follow the same trend as those produced by the atmosphere-based climate scenarios and we can assume that MODIS-derived environmental traits obtained for the year 2050 are reliable. No climate scenarios are available for the year 2030.

### Fitting the models with actual distribution data of ticks and vertebrates and obtaining projections from environmental scenarios

Several algorithms are available to obtain prediction maps of species distribution (presence/absence, not abundance) based on descriptive variables of the habitat. In this study, we used the algorithm MaxEnt (Maximum Entropy), the efficacy of which has been widely demonstrated [29–31]. Distribution models need to be "trained" with the known distribution of the species to be modeled, from which the combination of variables that define the probability of a species’ presence in space is obtained. The records with the coordinates of the tick *I*. *ricinus*, the reservoirs of *B*. *burgdorferi* s.l., and the hosts of the vector were obtained from previous data that were made publicly available [16, 32]. These coordinates were already subjected to quality control at the time of publication [16]. The complete overview of the distribution of *I*. *ricinus* is included in S1 Fig. Briefly, the original dataset contains about 14,000 records of questing *I*. *ricinus* in Europe, plus around 3 million records of the 160 species of vertebrates on which the ticks has been collected feeding. The original paper [32] introduced the concept of “contact rates” as the habitat overlap between the tick and vertebrates weighted by the relative importance of each vertebrate for supporting populations of the tick, or the relative importance of each host as reservoir of *Borrelia* spp. The original dataset also provided several scripts for the R programming environment [27] allowing calculations of expected distributions, niche overlapping and contact rates between the tick and the vertebrates expected to colonize every image pixel. All the models illustrating the predicted distribution of the vector and the vertebrates were validated and discussed previously [32]. The distribution dataset of vertebrates was obtained from GBIF, and is also publicly available [32].

We included 18 species of vertebrates. The species were chosen according to a recent review as being the most important in the circulation of either *B*. *garinii* or *B*. *afzelii* [33]. Mammalian species reported to be reservoirs of *B*. *afzelii* and the number of records over the target territory with reliable pairs of coordinates are: *Apodemus agrarius* (612), *Apodemus flavicollis* (2,180), *Apodemus sylvaticus* (10,287), *Erinaceus europaeus* (15,062), *Lepus europaeus* (17,709), *Microtus agrestis* (2,180), and *Myodes glareolus* (54,670). Bird species, the main reservoirs of *B*. *garinii*, are *Cyanistes caeruleus* (122,049), *Erithacus rubecula* (104,472), *Fringilla coelebs* (153,063), *Parus major* (99,476), *Phylloscopus collybita* (93,190), *Sylvia atricapilla* (23,347), *Turdus merula* (129,003), and *Turdus philomelos* (86,230). Other vertebrates included in the study that are not recognized reservoirs of *B*. *burgdorferi* s.l., but important hosts for *I*. *ricinus*, are *Capreolus capreolus* (29,807), *Cervus elaphus* (9,199), and *Vulpes vulpes* (49,492). Vertebrate records total 1,002,028 coordinate pairs and the distribution of *I*. *ricinus* is represented by 14,108 points.

To calculate the environmental niche of each species, the “wallace” package [34] for R [27] was used. This library depends on the MaxEnt algorithm to obtain the expected distribution under current conditions (years 2002–2017) and project it on future scenarios. As explanatory variables, the first three coefficients of the Fourier series for LSTD and NDVI were used (six variables in total). As indicated above in the section “obtaining climate data” the environmental variables describing the niche of the ticks and the vertebrates were derived from a Fourier transform (i.e. harmonic regression) of the monthly time series of satellite images. This procedure deconstructs the complete time series into coefficients that synthetically describe the original data. It has been demonstrated that this approach produces a better modelling outcome than using interpolated climate data or averaged monthly estimates directly derived from the time series [20]. These variables describe the mean value of the variable for the considerd period of time (either LSTD or NDVI), the slope in the spring (i.e. how fast or slow is the spring rise of LSTD or NDVI) and the negative autumn slope (i.e. how fast the summer turns into lower autumn values of LSTD or NDVI). The algorithm was trained with 50% of the records of each species to be modeled, using the remaining 50% to iteratively check the confidence of the fit. The whole process was repeated 10 times for each species, randomly selecting different training and test sets, obtaining the best possible distribution model. The suitability of each model was verified using the area under the curve (AUC) of the test set, as the Akaike information criterion provides erroneous results when applied to a geographical extension [35]. The AUC compares the outcome of each model with the recorded distribution of each species, producing an evaluation of its quality in terms of similarity of the predicted *vs* its known distribution.

Predictive raster maps were produced for each species and stacked to calculate the environmental overlap of the tick with each species of vertebrate. This is calculated on a pixel by pixel rate for the complete raster, comparing the values of the probability of presence of the tick and any other vertebrate at each pixel. The calculations of the contact rates among vertebrates and the tick were obtained using an already published R script that explicitly computes the habitat overlap between each species of reservoir, vertebrate, and the tick in the range of each environmental variable, producing a value reweighted between 0–100. The script is publicly available [32].

### Projection of results into the geographic space

All data resulting from the calculations above were summarized in the biogeographical regions of the target territory. We used the LANMAP2 product [36] that describes the biogeographic characteristics of the European territory. In addition to providing a coherent definition of the territory, LANMAP2 synthesizes the region into 14 climatic domains. This procedure resulted in maps that display the projections of contact rates into an easily captured geographic space.

## Results

**Fig 1:**
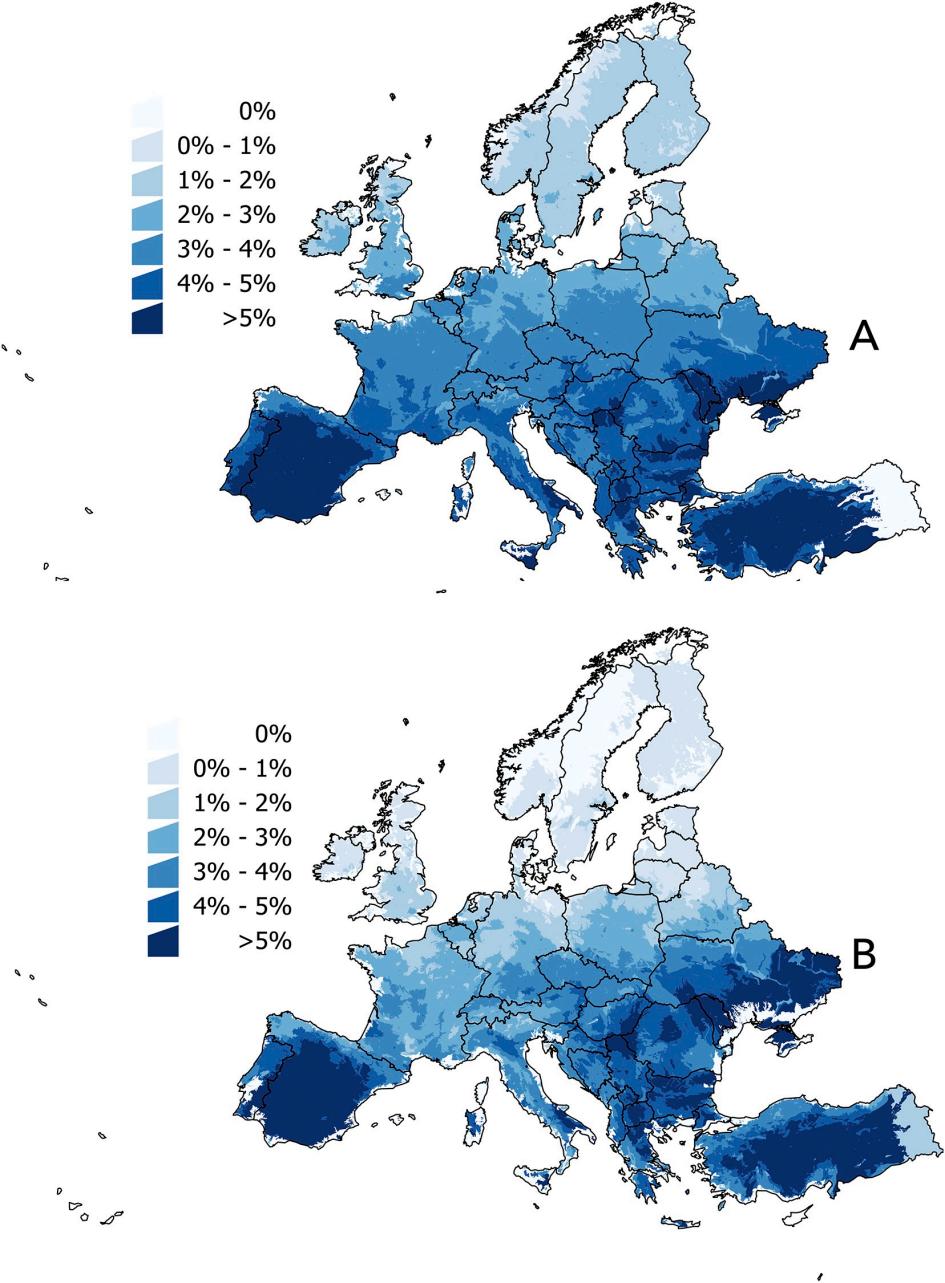
Difference (in %) between satellite-derived values of annual averaged land surface temperature and the interpolated annual averaged temperature obtained from WorldClim. (A) In the period 2002–2017. (B) In the year 2050.

The difference between the MODIS- and WorldClim-derived temperature had a relative narrow range, with a greater difference in southern latitudes (Fig 1). The pattern was similar in both the baseline period and the year 2050, with smaller differences at the later time period. A pixel by pixel comparison indicated a lack of significant differences between the discrepancies in temperature in both the the baseline period and 2050 (F = 1251.08, p = 0.156), indicating that MODIS-projections to 2050 do not significantly differ from purposely developed scenarios of interpolated climate. Therefore, the regression used to build the LSTD and NDVI values for 2030 and 2050 was assumed to be realistic. No scenarios exist for 2030.

**Fig 2:**
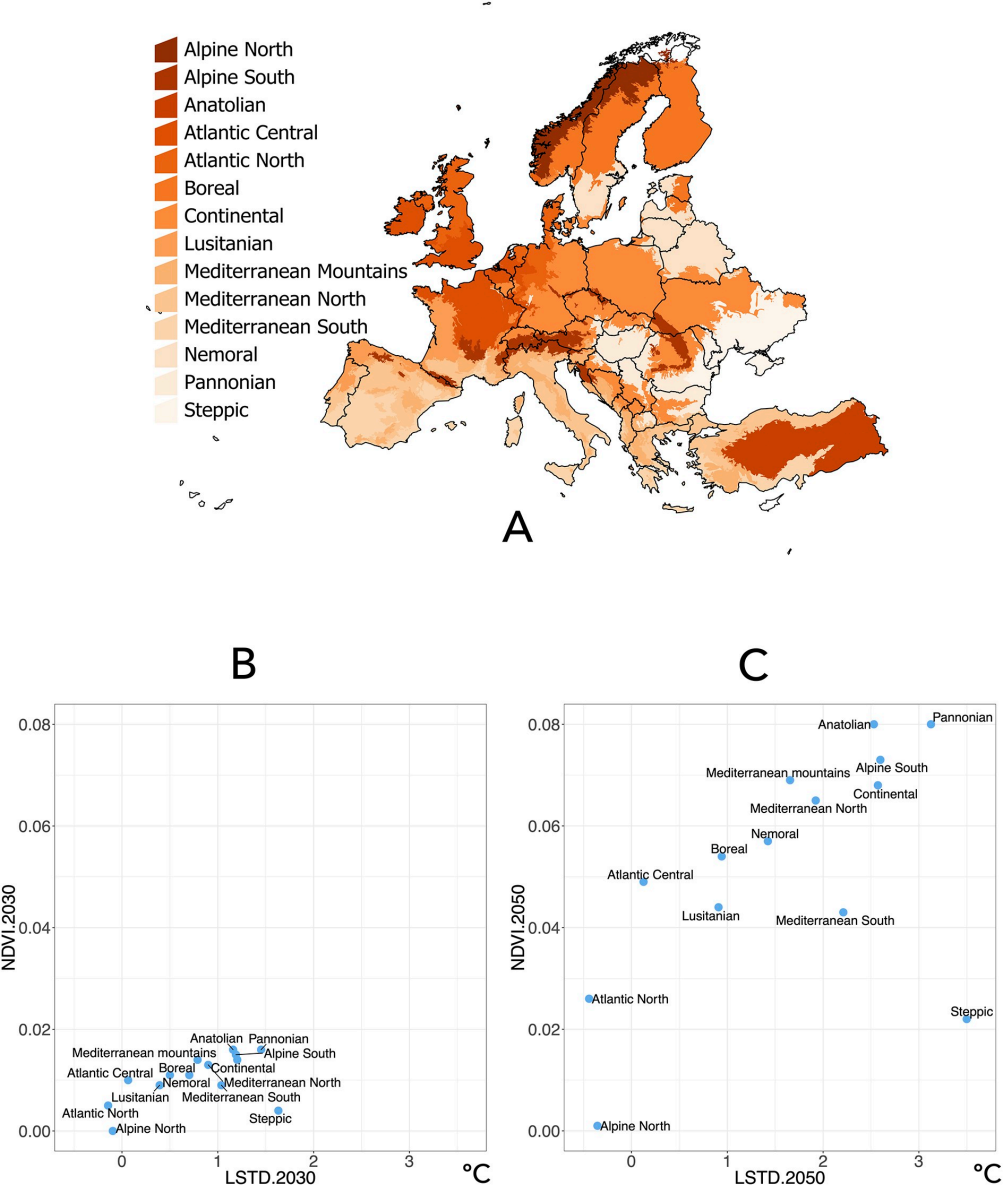
The biogeographic regions of Europe used in this study with the trend of environmental traits. (A) The geographic distribution of the biogeographic regions used in this study based on LANMAP2. (B) The projected changes in the values of the satellite-derived land surface temperature (LSTD) and the Normalized Difference Vegetation Index (NDVI) in the year 2030 compared to the baseline period (2002–2017) and obtained by time series regression. (C) The projected changes in the values of the LSTD and NDVI in the year 2050 compared to the baseline period (2002–2017) and obtained by time series regression. NDVI values are unitless and range from -1 to +1.

**Fig 3:**
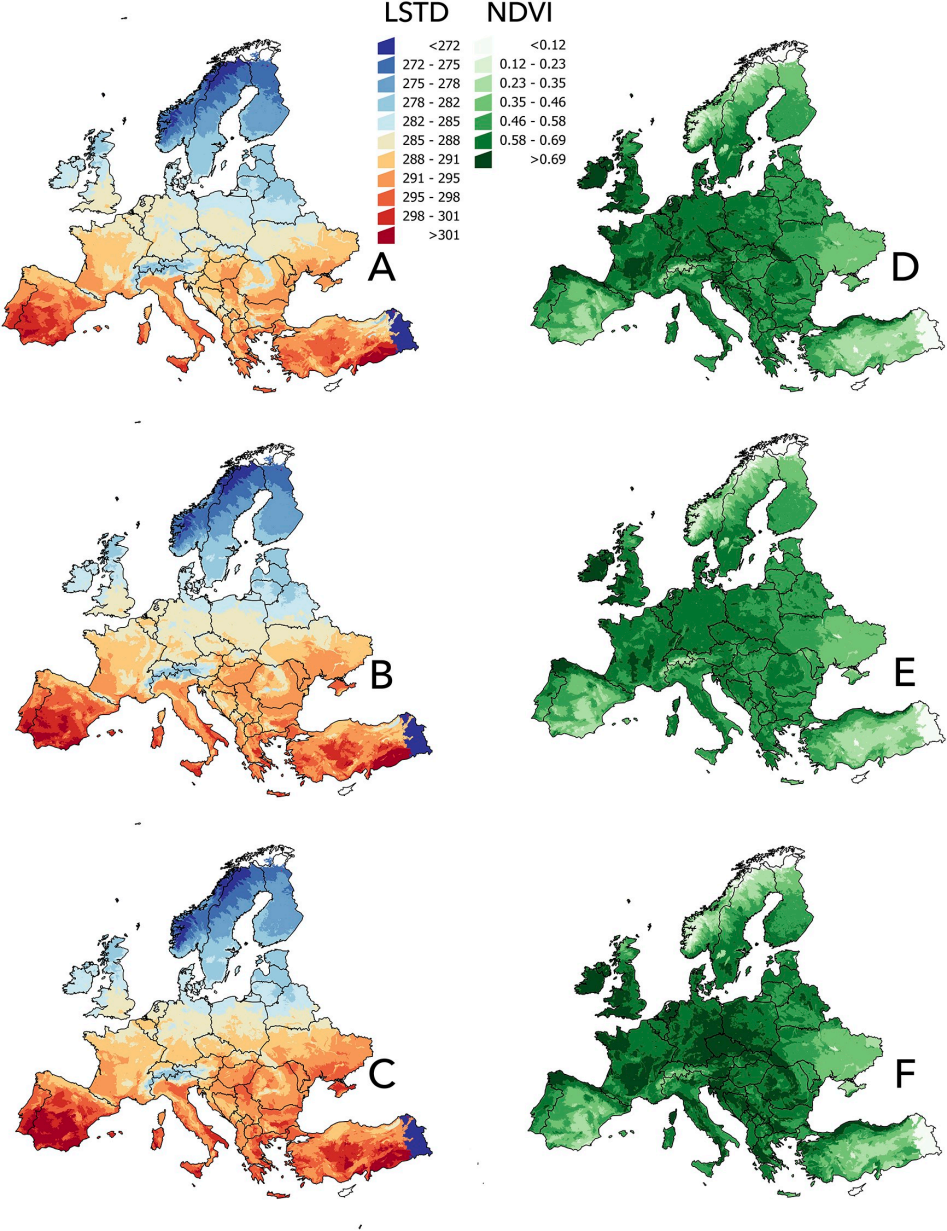
Spatial projection of the satellite-derived annual average land surface temperature and Normalized Difference Vegetation Index (NDVI). (A) Land surface temperature in the periods 2002–2017, (B) 2030, and (C) 2050. (D) NDVI in the periods 2002–2017, (E) 2030, and (F) 2050. Values of temperature are in Kelvin; values of NDVI are unitless.

Fig 2 shows the trends in LSTD and NDVI aggregated for the European biogeographic regions extrapolated for the years 2030 and 2050 with respect to the baseline values. In general, all biogeographic regions undergo projected changes of variable magnitude, with an obvious trend in the years 2030 and 2050: increases of up to 0.5°-1° in LSTD and increases in the NDVI of 0.02–0.08 (on a scale of -1 to 1). Note the changes in the Lusitanic, Boreal, Nemoral, Southern and Northern Mediterranean, Mediterranean, Continental, Southern Alpine, Anatolian, and Panonic regions. The climate determined for the two time periods is projected to be moderately warmer, with a longer vegetative period (growth season). An exception to this are the southern areas, which would experience a greater projected temperature increase, promoting a decrease in the green period (or higher dryness). Fig 3 projects these forecasts on the geographic context.

**Fig 4:**
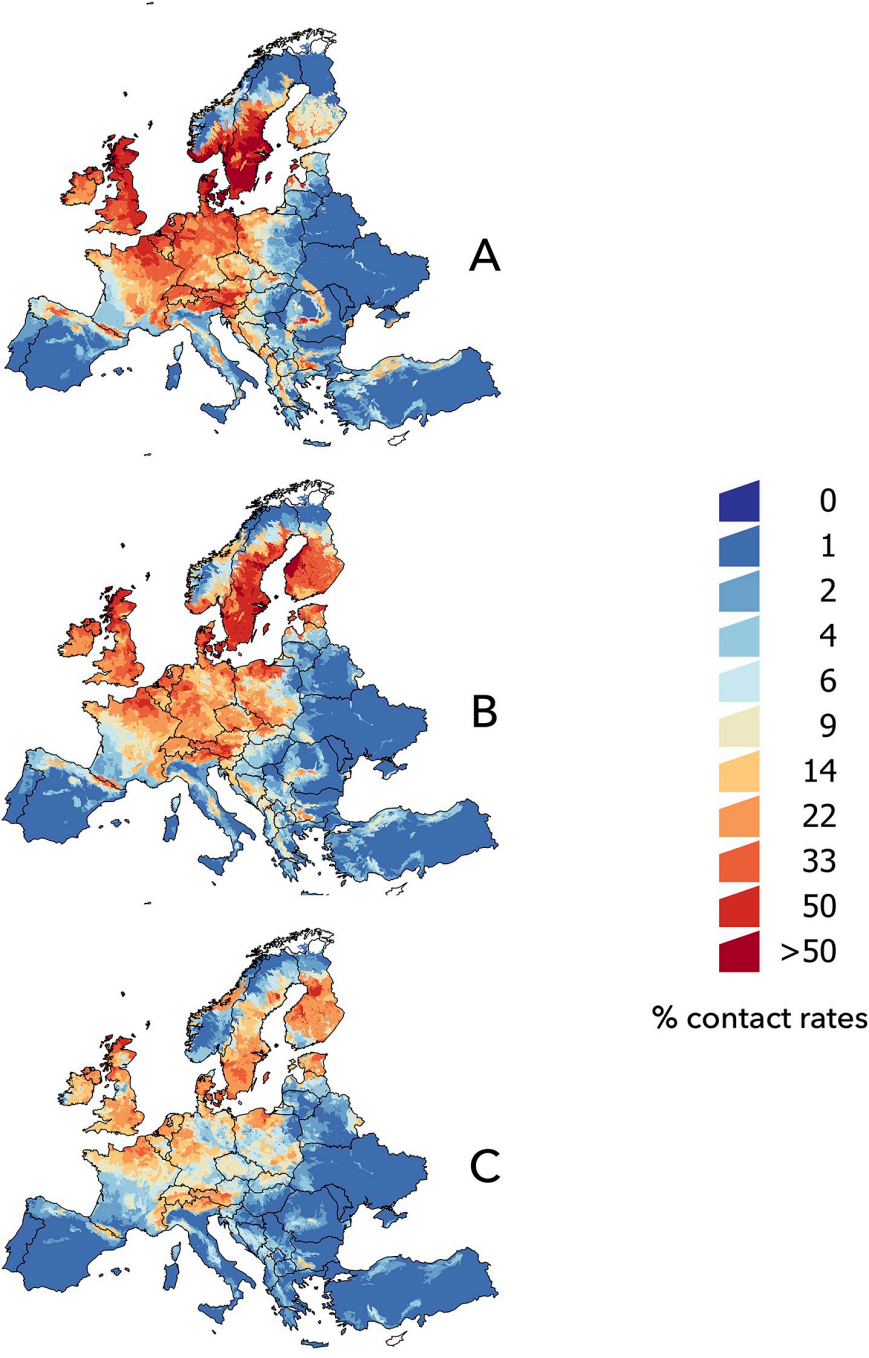
The spatial distribution of the projected contact rates between *Ixodes ricinus* and avian reservoirs of *B*. *garinii* in the target territory. (A) 2002–2017, (B) 2030, (C) 2050.

The raw distribution maps of every species had an AUC > 0.72 in every case (minimum for *Apodemus sylvaticus*, 0.72; maximum for *Ixodes ricinus*, 0.92). We do not intend to explain the behavior of each individual model for each species and how organisms use the environmental niche; our goal was to obtain the contact rates and track their trends under climate conditions expected in the near future. The predicted environmental suitability weighted by the probability of contact between *I*. *ricinus* and bird species for the different slices of time is included in Fig 4. In the maps, warmer colors indicate a greater probability of habitat overlap between the vector and reservoirs, which is interpreted as a higher risk of pathogen circulation.

The climate in the baseline period (Fig 4A) reflects a wide co-distribution of the vector and bird species throughout central Europe, the British Isles, southern Norway, and wide regions of central and southern Sweden. Note the wide area of high probability of contact among birds and *I*. *ricinus* in the strip of the Mediterranean coast of the Balkan countries. The projection of climate data to the year 2030 (Fig 4B) predicts an increase in vector-bird contact in a large territory of southern Finland and Estonia, an increase in latitude on the coast of Norway, and increased risk in eastern areas of the European territory, such as Poland, the Czech Republic, and Slovakia. However, climate projections for the year 2050 predict a decrease in areas in which the vector and birds could coexist (Fig 4C). Forecasts for that slice of time still delineate the linear relationship between contact rates and latitude. A patent decrease in the probability of overlap among birds and *I*. *ricinus* is predicted in the rest of Europe. In short, contact rates among *I*. *ricinus* and the selected species of birds would reach a maximum around the year 2030, and then decrease as the LSTD and NDVI traits promote a decrease in the probability of contact among the vector and bird reservoirs.

**Fig 5:**
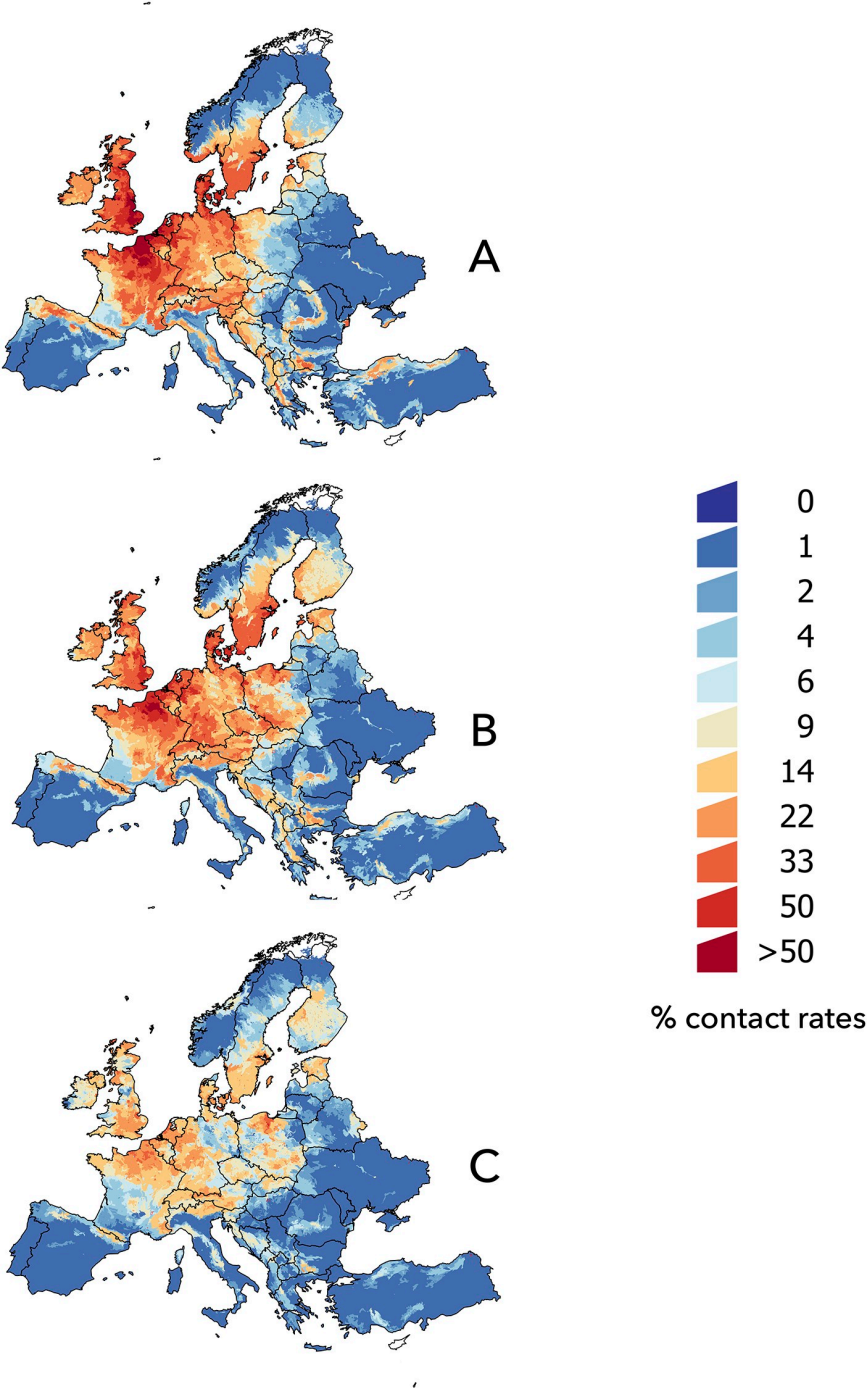
The spatial distribution of the projected contact rates between *Ixodes ricinus* and mammalian reservoirs of *B*. *afzelii* in the target territory. (A) 2002–2017, (B) 2030, (C) 2050.

The modelling of the habitat sharing among *I*. *ricinus* and the mammalian reservoirs of *B*. *afzelii* (Fig 5) shows clear differences from the predictions regarding birds and *B*. *garinii*. The spatial projection shows wide areas of high contact rates along the Atlantic regions, central Europe, and southern Scandinavia (Fig 5A). The trend for the year 2030 predicts that changes in contact rates over time are smaller than those previously noted for birds, with a northern and eastern trend (Fig 5B). Changes were predicted mostly in the Baltic countries, areas of Eastern Europe, southern Finland, and the Balkans. Models projected into the year 2050 (Fig 5C) show a general decrease in the probability of overlap between the vector and mammalian reservoirs, though at some points, such as in Finland, the probability of contact is still higher than in the baseline period.

**Fig 6:**
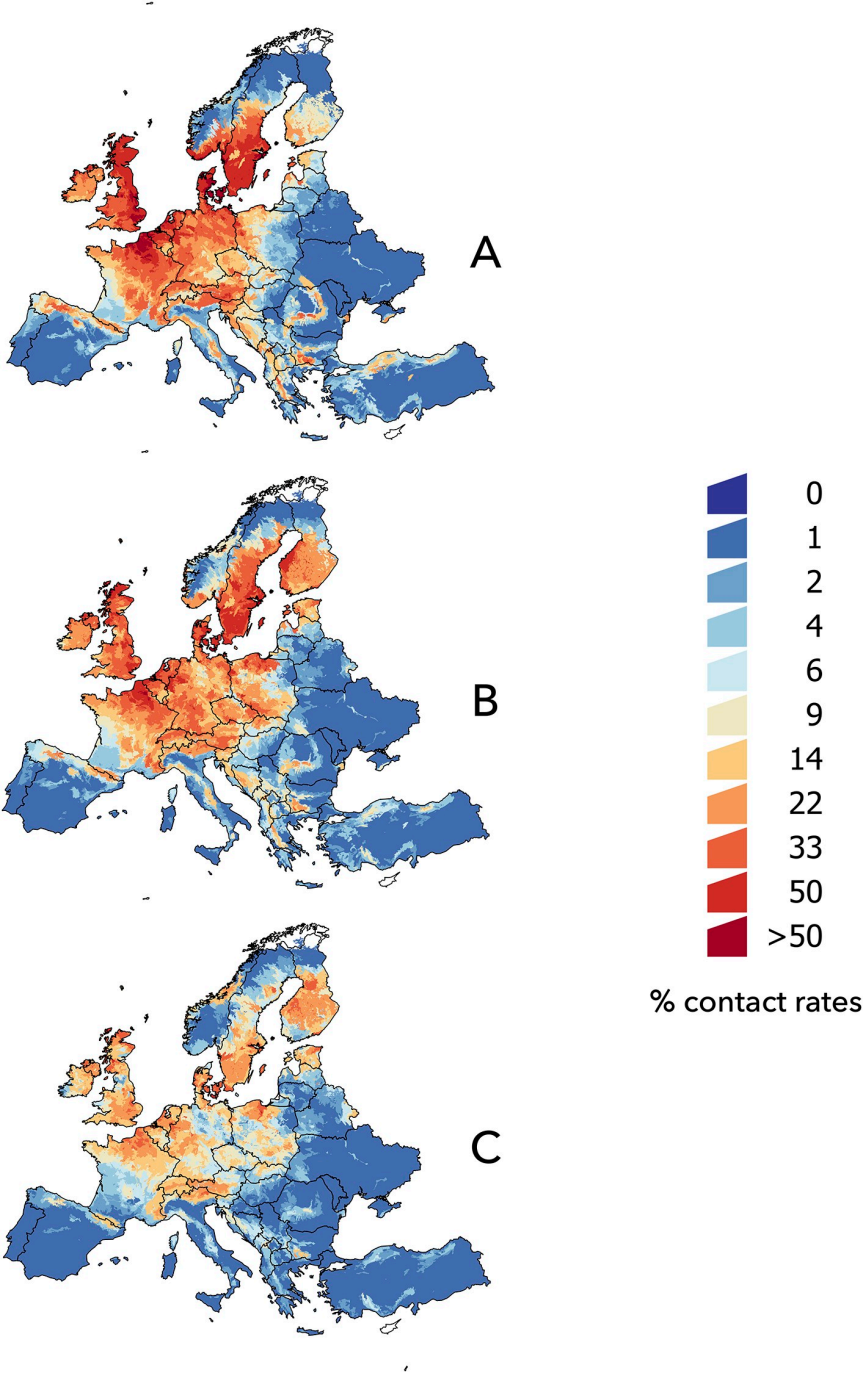
The spatial distribution of the projected contact rates between *Ixodes ricinus* and the mammalian hosts that are not reservoirs of *Borrelia* spp. (A) 2002–2017, (B) 2030, (C) 2050.

The modelled contact rates among *I*. *ricinus* and some non-reservoir hosts for the baseline period (Fig 6A) show similar predictions; maximum environmental overlap is forecasted along the Atlantic coast and in the south of the Nordic countries. The models projected into the years 2030 and 2050 (Fig 6B and 6C) predict a general decrease in the overlap between *I*. *ricinus* and some of its main hosts in the lower latitudes, together with an increase at higher latitude and longitude in the target territory.

## Discussion

We addressed how climate trends could impact the relationships among *I*. *ricinus*, the reservoirs of *B*. *burgdorferi* s.l. in Europe, and other vertebrates that feed the tick to capture the probable contact rates of organisms circulating the pathogen. The analysis is based on previous developments that refer to the environmental niche as the basic method for evaluating these relationships [14,32]. Tick-borne pathogens persist due to a strict combination of environmental variables, which model the distribution of reservoirs that allow their circulation in permanent foci [25]. The novelty of this approach lies on the well supported concept that stable foci of a tick-borne pathogen require not only adequate environmental conditions for the vector, but also a sufficient contact rate with reservoirs [37]. Although mechanistic models have been used for years to delineate the probable distribution of ticks, they had not been used together with the expected habitat overlapping with reservoirs of a tick-borne pathogen, shaping the areas in which the pathogen could circulate. Previous modelling approaches on the impact of climate trends on ticks and risk of Lyme borreliosis addressed the changes of the life cycle of the tick under the climate conditions of future scenarios [5,13]. A recent study with similar aims [17] considered Lyme borreliosis risk driven by the presumed abundance of the vector and an estimation of the habitats preferred by vertebrates (without considering particular species), evaluating the expected changes under the conditions of future climate scenarios. That study [17] predicted that an enlarged period of tick development could lead to increased rates of transmission of *Borrelia* spp. These modelling approaches, however, look unrealistic since the “current” scenario predicted a high risk by Lyme borreliosis at peak season in areas where the tick is absent (i.e. eastern Spain) or a lack of risk in endemic countries (i.e. Ireland). In the current study, we claim that mechanistic models based on the environmental niche of the target tick, its hosts, and the *known* reservoirs of a pathogen could unveil both biotic and abiotic effects derived from climate trends, complementing process driven models [13] and producing a pixel-size picture of the presumed distribution of each organism involved in the circulation of *Borrelia* sp. Further on this, modelling separately two species of *Borrelia* with known different reservoirs provided a better understanding of the climate actions impacting the relationships between vectors and reservoirs.

This framework is based upon an explicit environmental description provided by satellite images and harmonic regression [38]. Time series of satellite data can be deconstructed into their harmonic components, which are not autocorrelated and explain the climate of a time period with a few variables. The use of interpolated climatologies [39] may be unreliable in this context because of the spatial autocorrelation of the descriptive variables, which artificially increases the statistical reliability of the models; the lack of ecological significance of some variables for ticks; the interpolated climatologies describe temperature in the air, whereas ticks live on the ground; and the lack of estimations of relative humidity or saturation deficit [40], which were replaced by the NDVI in our approach. The NDVI represents the photosynthetic vigor [41] that has been shown to be correlated with *I*. *ricinus* tick distribution.

The primary source of uncertainty in the current study arose from the reliability of the projected climate for 2030 and 2050. We obtained estimations of the near future climate by applying a time series regression to the baseline period of averaged monthly data (2002–2017). We acknowledge that linear regression may not completely capture the climate trends in such a large territory, as they depend on complex atmospheric interactions. Therefore, we opted to compare whether the deviations from other temperature datasets were of similar magnitude between two time periods (baseline and 2050), indicating that our projections are valid. An ANOVA unveiled a lack of significant differences between the deviations of both sets of values (baseline and 2050). Therefore, MODIS-projected values for both LSTD and NDVI in 2050 were considered reliable and robust enough to proceed.

A secondary source of uncertainty arose from the reliability of the distribution modeling of the tick and species of vertebrates that interact, producing contact rates, which depend on the extent to which explanatory variables influence the model outcomes. Every model was within the expected margins of reliability, with AUC values well over 0.7 (range 0–1). As explanatory variables are not autocorrelated, which is a feature that affects model performance [20], we consider AUC an adequate marker of model reliability. In addition, the high number of coordinates for vertebrates and the best existing *I*. *ricinus* dataset (more than 14,000 records, collected by specialists) convey reliability to the modeling approach.

The current study only considered the relative contributions of hosts acknowledged to have a recognized competence in the circulation of *Borrelia* spp. [33] disregarding other vertebrates that may have a local impact in the target territory. It also unaddressed features regarding the relative impact of density of vertebrate populations (i.e. deer or other wild ungulates *versus* rodents or birds) on the abundance of the tick or the persistence of *Borrelia* spp. Therefore, an unaddressed source of uncertainty depends on the relative faunal composition at the landscape scale and deserves further research within the framework outlined in this study. While we included the *most* important reservoirs of *Borrelia* spp. in the target territory, as reported [14, 33], the current study did not consider *every* possible reservoir. In some cases, it has been because the lack of a reliable number of coordinates for modelling; in others because no adequate data on reservoir abilities have been obtained. Undoubtedly, the exclussion of vertebrates that may be abundant at the local scale would bias predictions. However, such a bias should be negligible at the scale of biogeographical region, as addressed in the current study. In any case, if the proposed framework is transferred into a higher resolution, special attention to this source of uncertainty should be spacially paid.

The general overview of the expected climate shows a trend towards higher LSTD and NDVI values, with the exception of southern regions of Europe, in which the NDVI shows a tendency for higher aridity. Most important in the context of this study is the increase in LSTD and NDVI in regions of Eastern Europe and Scandinavia. The clear trend of increased NDVI in the Nordic countries is a point of interest in the epidemiology of Lyme borreliosis. Our projections are in line with previous conclusions regarding the movement of Lyme borreliosis to northern latitudes as a consequence of a longer growth season [42]. There is serious concern regarding the spread of *B*. *burgdorferi* s.l. into the northern latitudes in Europe [43]. This is not only the consequence of a northern spread of *I*. *ricinus*, which has already been recorded at 65°N [10], but also of the joint spread of both the tick and the reservoirs of *Borrelia* spp., together with local high densities of large (non-reservoir) mammals supporting the adult ticks. The modeled forecasts agree with available field data, underlining the suitability of this modeling approach to explicitly shape the habitat overlap.

Both *B*. *garinii* and *B*. *afzelii* have already been reported to have a different distribution in Europe, which is attributed to their reservoirs’ different responses to the environment [37]. This is a point of interest because the climate trends, at least for the years 2030 and 2050, are predicted to promote a divergence in the distribution of reservoirs, with a direct impact on the probable distribution of the pathogens. The projections for the year 2030 show a dramatic potential spread of *B*. *garinii* in large northern areas, some parts of Central Europe, and the Baltic countries. *Ixodes ricinus* has traditionally been restricted in the eastern European region by colder winter temperatures. In these areas, *I*. *ricinus* is replaced by *I*. *persulcatus*, extending through Russia to northern Japan [44]. Field studies on the dispersion of *B*. *garinii* towards northern latitudes [42] support a correlation between a longer growth season and the northern spread of *Borrelia* spp., but the spatial pattern of *B*. *afzelii* has not been explored in depth. Our forecasts correspond well with actual data on the prevalence of these bacteria in Europe [37] and support that modeling based solely on the impact of climate on the vector [17, 37] is insufficient for capturing the range of tick-borne pathogens if the distribution of hosts and reservoirs is disregarded. Our models highlight that climate trends could contribute to split the range of both species of bacteria. However, this trend could be interrupted under the climate conditions projected into 2050. The estimations for that period could disrupt the circulation of the pathogen, although isolated foci would persist. This was an unexpected result. Our estimations predict a clear disruption of the habitat sharing among the reservoirs of a pathogen and its tick vector. This does not mean an extinction of *I*. *ricinus* because of extreme environmental conditions, but a fracture of the finely tuned environmental overlap among reservoirs and the tick that drives the persistence of *Borrelia* spp. foci.

The capture of environmental suitability for the vector of an infectious agent, its hosts, and reservoirs over large areas is a direct, ecologically meaningful method for generating scenarios that allow epidemiological decisions to be made. Satellite pictures have enormous power to describe abiotic traits impacting ticks and the reservoirs of tick-borne pathogens. As presented here, this background cannot yet capture the high-resolution habitat fragmentation, which may generate the foci observed in the spatial distribution of Lyme borreliosis [45]. However, it is a promising tool that may supersede other approaches, providing comprehensible insight into the scenarios of future climate conditions if adequately trained. The unexpected predictions regarding the decrease in habitat sharing among *I*. *ricinus* and vertebrates around the year 2050, and the consequent decrease in pressure by some infectious agents deserves further research.

## Supporting information

S1 Fig The recorded distribution of *Ixodes ricinus* in the target area. The figure includes about 14,000 records of all the stages of *I*. *ricinus* with adequare georeferencing. The vast majority of the records have been validated by local experts. However, part of the records in Finland are old (around 1970’s) and would need to be re-examined. Some records of the tick in Africa may represent a different species, *Ixodes inopinatus*. All the data are available at https://datadryad.org/resource/doi:10.5061/dryad.2h3f2.
(PDF)
