# Supplementary figures and images for: Could climate trends disrupt the contact rates between Ixodes ricinus (Acari, Ixodidae) and the reservoirs of Borrelia burgdorferi s.l.?
Source: PLoS One. 2020 May 29;15(5):e0233771. doi: 10.1371/journal.pone.0233771 (PMC7259620; doi:10.1371/journal.pone.0233771)

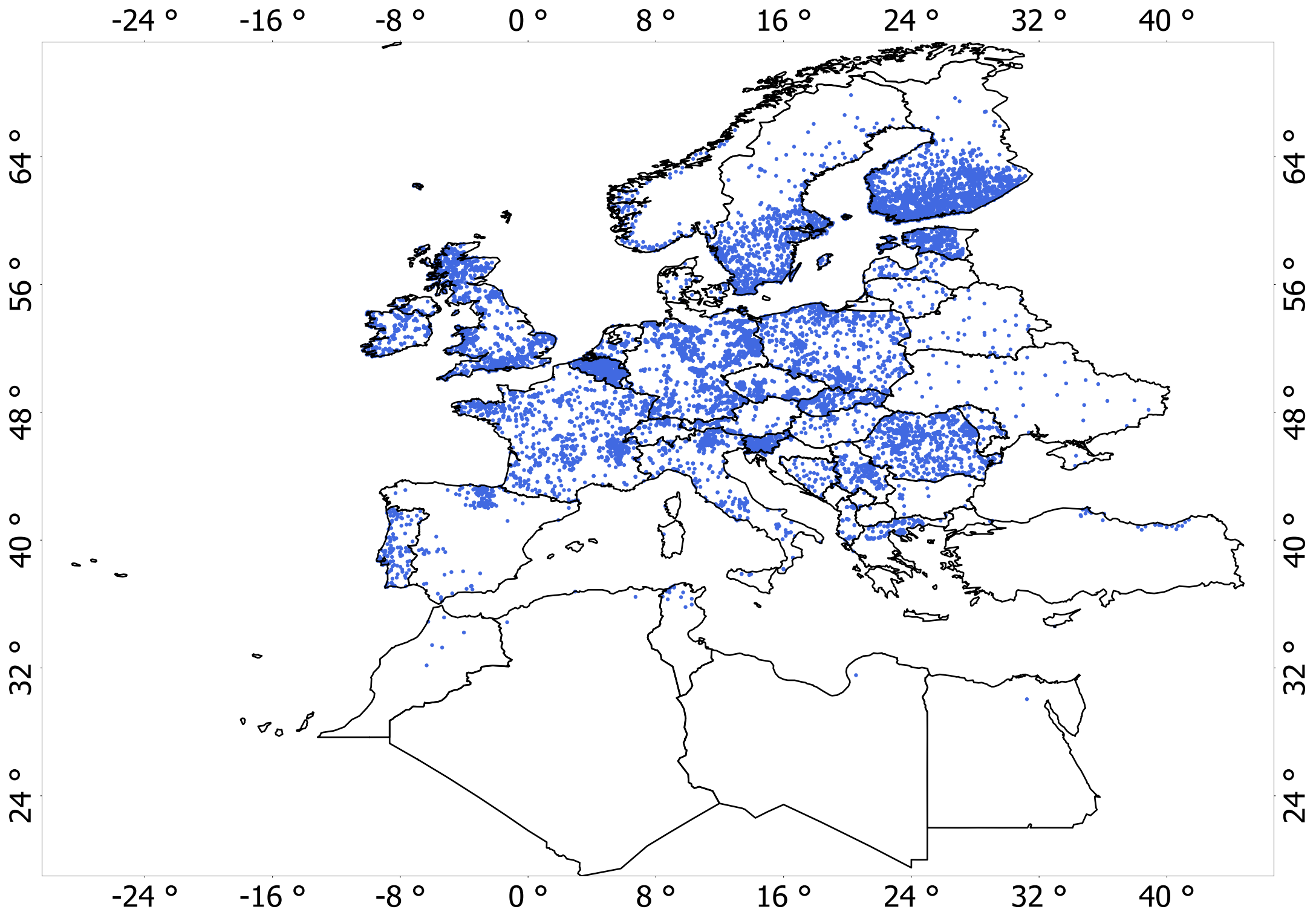

Supplement: S1 Fig — The figure includes about 14,000 records of all the stages of I. ricinus with adequare georeferencing. The vast majority of the records have been validated by local experts. However, part of the records in Finland are old (around 1970’s) and would need to be re-examined. Some records of the tick in Africa may represent a different species, Ixodes inopinatus. All the data are available at https://datadryad.org/resource/doi:10.5061/dryad.2h3f2. (PDF) [file pone.0233771.s001.pdf]
